# NIRS in motion—unraveling the neurocognitive underpinnings of embodied numerical cognition

**DOI:** 10.3389/fpsyg.2014.00743

**Published:** 2014-07-18

**Authors:** Julia Bahnmueller, Thomas Dresler, Ann-Christine Ehlis, Ulrike Cress, Hans-Christoph Nuerk

**Affiliations:** ^1^Department of Psychology, University of TuebingenTuebingen, Germany; ^2^Knowledge Media Research CenterTuebingen, Germany; ^3^LEAD Graduate School, University of TuebingenTuebingen, Germany; ^4^Department of Psychiatry and Psychotherapy, University of TuebingenTuebingen, Germany

**Keywords:** embodied cognition, functional near-infrared spectroscopy (fNIRS), numerical cognition, motion, imaging techniques

The central representation of numerical cognition is commonly considered an abstract magnitude representation serving as one key precursor for higher mathematical thinking. However, recent research indicates that the representation might not be purely abstract. In fact, accumulating evidence suggests that numerical representations are rooted in and shaped by specific motor activities and sensory-bodily experiences and, therefore, are influenced by so-called embodied numerical representations. If we want to understand how numerical understanding develops, it is crucial to elucidate the basic cognitive tools with which we develop a sense of number. We argue that it is necessary to address this issue on both a behavioral and a neural level.

Contrasting the view of functional magnetic resonance imaging (fMRI) being the generally preferable neuroimaging technique, we argue that particularly in embodied cognition, restrictions and benefits of different imaging methods should guide the chosen research question. In our opinion, near-infrared spectroscopy (NIRS) is optimally suited to investigate embodied cognition paradigms that explicitly involve motion. In the following, recent research will be outlined showing that numerical cognition is not purely abstract, but influenced by embodied representations. NIRS will then be introduced as a feasible technique for the investigation of embodied cognitions. Since research in this domain is largely restricted to the perception of embodied experiences, but fails to address motion itself, we will finally argue that NIRS offers a good opportunity to fill this research gap.

## Embodied numerical cognition: where we are

Embodied cognition refers to the idea that, throughout our lifespan, we consistently associate specific motor activities and sensory-bodily experiences with more abstract concepts such as words or numbers (Barsalou, 2008). In numerical cognition, a growing body of research indicates that number is a prime example of such embodied cognitions. To clearly separate embodied numerical cognition from other related concepts influencing the way we learn, represent and deal with numbers, Fischer (2012; Fischer and Brugger, 2011) distinguishes grounded, situated and embodied numerical cognition. Grounded numerical cognition means that universal laws of the physical world are reflected in our representation of numbers (i.e., small numbers are associated with lower space whereas large numbers are associated with upper space). Situated numerical cognition refers to the idea that situations (including external stimuli as well as our body posture) influence how we process numbers. In this vein, Loetscher et al. (2008) demonstrated that turning the head to the right resulted in the production of larger random numbers than turning it to the left. In contrast, embodied numerical cognition relates to repeated, culturally dependent learning experiences directly associating representations of number with specific motor activities or other bodily-sensory experiences.

Several research branches in embodied numerical cognition have addressed questions of automaticity, directionality, functionality, developmental aspects as well as the generality of embodied numerical representations on behavior. A rather concrete link between number magnitude and embodied numerosity is investigated in the most prominent example of embodied numerical cognition: finger counting. There is behavioral evidence that the activation of this association is (i) automatic (e.g., Klein et al., 2011), (ii) already evident in childhood (Domahs et al., 2008), and persisting into adulthood (e.g., Di Luca et al., 2006; Klein et al., 2011). Furthermore, the association is (iii) culturally dependent (e.g., Domahs et al., 2010), and (iv) dependent on the spatial representation of numerical magnitude (cf. the mental number line; e.g., Fischer, 2008; Lindemann et al., 2011). A second branch supporting and generalizing findings from finger counting is grasping. Research on grasping shows that the association between the number magnitude representation and grasping actions is also (i) automatic (Andres et al., 2004, 2008; Lindemann et al., 2007; Ranzini et al., 2011) and additionally (ii) bidirectional, meaning both that the representation of number magnitude influences grasping actions (e.g., Andres et al., 2004; Badets et al., 2007) and vice-versa (Badets and Pesenti, 2010; Badets et al., 2010). It is important to note that, with few exceptions, behavioral studies on finger counting and grasping do not include actual actions except for response giving. Rather, the association of numerical magnitude and fingers/hands is achieved by perceptual and mostly static cues (e.g., finger postures on a screen). This does not mean that these studies do not give important insights into the association of number magnitude and finger-/hand-based representations. However, questions of how and why the association develops, how and where it originates and whether or not it is influenceable remain open.

To investigate the functional relevance, generality and variability of associations of number magnitude and embodied representations, first correlation and training studies have been conducted. Concerning finger counting, finger-based representations are functionally relevant for numerical development: Noël (2005) showed that finger gnosis predicts future numerical skills. Moreover, a systematic training of finger gnosis ameliorated numerical performance (e.g., Gracia-Bafalluy and Noël, 2008).

Few studies investigating the neurocognitive underpinnings of embodied numerical representations are available. Usually, neural correlates (the “where”) of associations of finger- as well as hand-related and numerical magnitude representations have been studied in perceptual studies. FMRI studies demonstrated that cortical areas related to number magnitude processing are in close proximity to areas activated when fingers/hands are used. Additionally, it has been shown that applying transcranial magnetic stimulation (TMS) to the left angular gyrus resulted in a disruption of finger schema and number processing (e.g., Rusconi et al., 2005). Evidence for automatic activation of fingers when dealing with numbers comes from an fMRI study (Tschentscher et al., 2012). They found an automatically coactivated right motor cortex when small numbers were passively viewed by subjects habitually starting finger counting with the left hand. Developmental changes were indicated by Kaufmann et al. (2008) showing that the coactivation of finger- and number-related areas is more pronounced for children as compared to adults. Taken together, similar to behavioral results, neural evidence supports the existence and automaticity of an association of finger-/hand-based and numerical magnitude representations. However, mostly perceptual and static cues have been used, which prevents direct insight into questions of how and why the association is established, how it originates and whether or not we can or should promote this association to increase learning outcomes. Moreover, the assumption that findings from perceptual paradigms can be generalized to actually motion-involving paradigms still needs to be tested.

## Spatial full-body movements subserving abstract semantic representations

There are first studies showing that not only finger but also body movements corresponding to the spatial representation of numbers influence number processing. Here, it is important to keep in mind that every bodily movement is accompanied by a spatial processing component (e.g., whenever we turn our head in a certain direction, we not only have a bodily-sensory experience but also a spatial one). Following the idea that the coactivation of a corresponding embodied-spatial representation of number might promote the development of the numerical magnitude representation, training studies were designed aiming at enriched training conditions that use (full) body movements and are congruent with a left-to-right orientation of the mental number line. Indeed, first studies indicate that such embodied-spatial numerical trainings are more effective than non-embodied control trainings and even show specific transfer effects (Fischer et al., 2011; Link et al., 2013; for an overview, see Moeller et al., 2012). This suggests that embodied numerical cognitions are not restricted to fingers/hands but generalize to more complex bodily experiences that relate to the spatial representation of numerical magnitude. Furthermore, it indicates that we can and should use the association of number and embodied-spatial representations to support mathematical learning, meaning that we should use motion and bodily-sensory experiences that correspond to the semantic and spatial representation of numerical magnitude. However, as motion can hardly be executed using the most prominent imaging methods, the neural mechanisms of how the association is established and of how learning is supported by such an enriched learning environment has not yet been addressed. Here, NIRS represents a good opportunity to begin filling this research gap.

## Imaging methods and motion

Several different neuroimaging tools are available, each with obvious benefits, but with specific shortcomings as well. Therefore, when choosing one of them, the respective research question needs to be considered. For instance, fMRI with its high spatial resolution enables detailed insights into the function of both superficial and deep neural structures. Electroencephalography (EEG) with its high sampling rate allows for a precise investigation of temporal processes. Both are prone to motion artifacts and rely on a rather restrictive measurement setting—not a major problem for most paradigms, but an enormous limitation for some. Therefore, the investigation of processes inherently relying on motion or body postures remains a challenge. To address research questions that involve motoric and sensory (embodied) processes underlying seemingly abstract numerical cognition, we argue that NIRS is a good alternative.

NIRS measures cortical oxygenation by emitting near-infrared light to the brain using optical properties of oxy- and deoxygenated hemoglobin, thus providing an optical blood-oxygen-level dependent (BOLD) signal (Obrig and Villringer, 1997; Ferrari and Quaresima, 2012). Light emitters and detectors are attached to the head in various arrangements resulting in a grid of measurement channels. NIRS as neuroimaging method has been introduced two decades ago and was applied in various settings since (e.g., Ehlis et al., 2014). It is non-stationary and applicable as bedside technique and in other real-word settings (e.g., class room, Dresler et al., 2009). NIRS allows the measurement of subjects in more natural positions and under several body postures. For instance, one well-known study compared apple peeling with NIRS to mock apple peeling in an fMRI-fNIRS setting (Okamoto et al., 2004). It could be shown that the natural action resulted in a different activation pattern as compared to the mocked action, illustrating the feasibility of NIRS as an imaging device that can easily be used during daily activities as well as embodied cognition paradigms.

## Embodiment and NIRS: a next step

Research in embodied numerical cognition has addressed different research questions to show that embodied numerical representations do influence number processing and numerical learning. Nevertheless, it mostly focused on paradigms that do not include actual and intended motion but rather static, perceptual cues. On a neural level, we argue that NIRS—despite its sub-optimal spatial resolution—is especially suited to help filling this gap. So far, studies successfully using NIRS have been conducted both for different motor tasks (for an overview see Leff et al., 2011) and in the field of numerical cognition investigating different paradigms with adults (e.g., Richter et al., 2009; Cutini et al., 2014) and children (e.g., Dresler et al., 2009; Hyde et al., 2010). Combining those research branches, NIRS offers the possibility for examining embodied numerical representations as it allows measuring brain activity during natural movements and in ecologically more valid settings. Integrating online measures of embodied numerical representations can add to a more elaborated picture of the neurocognitive underpinnings of the interplay, origin and development of abstract and embodied numerical representations.

We are aware of existing problems that need to be addressed in NIRS methodology. Although NIRS is less prone to movement artifacts as compared to EEG and fMRI, it does not mean that it is not affected at all. When the head is moved, small agitations of the sensors reduce data quality as direction of light flow is changed. Different analytic approaches have been suggested and are used in practice to deal with movement (see Cui et al., 2010; Brigadoi et al., 2014). Furthermore, in recent years, remote NIRS devices have been developed which have already been applied during bicycle riding (Piper et al., 2014) and allow for greater freedom of motion than the common non-remote systems.

Additionally, NIRS has a low spatial resolution (both lateral resolution and penetration depth) depending on the distance between optodes. Therefore, studies asking for a fine-grained topological resolution of a particular region (e.g., Harvey et al., 2013) can currently not be investigated using NIRS. Unraveling the neuronal basis of higher cognitive processes such as (embodied) numerical cognition does, however, not solely rely on spatially high resolving devices and research questions. Nonetheless, ongoing methodological progress is made in overcoming shortcomings in terms of spatial resolution (for an overview see, e.g., Ferrari and Quaresima, 2012; Scholkmann et al., 2014). In terms of lateral resolution, already available high-density arrangements offer a much higher resolution when compared to broadly used continuous wave systems. Considering penetration depth, continuous wave systems only allow for measuring cortical structures located few centimeters under a respective emitter-detector-channel. Improved depth penetration can be achieved by frequency- and time-domain instrumentation allowing for a clearer separation between extra- and intracerebral oxygenation changes. Methodological research points in a promising direction and we are convinced that the availability of these higher-resolution devices will increase in the next years and will enlarge the feasibility of NIRS even further.

Considering new evidence in research in embodied numerical cognition as well as technological developments, we are convinced that NIRS will add to a more elaborated picture of neurocognitive underpinnings of embodied cognition and to a broader understanding of the basis of numerical cognition as well.

### Conflict of interest statement

The authors declare that the research was conducted in the absence of any commercial or financial relationships that could be construed as a potential conflict of interest.
